# A PATO-compliant zebrafish screening database (MODB): management of morpholino knockdown screen information

**DOI:** 10.1186/1471-2105-9-7

**Published:** 2008-01-07

**Authors:** Michelle N Knowlton, Tongbin Li, Yongliang Ren, Brent R Bill, Lynda BM Ellis, Stephen C Ekker

**Affiliations:** 1 Department of Genetics, Cell Biology and Development, University of Minnesota, Minneapolis, MN 55455, USA; 2 Department of Neuroscience, University of Minnesota, Minneapolis, MN 55455, USA; 3 Department of Laboratory Medicine and Pathology, University of Minneapolis, Minneapolis, MN 55455, USA; 4 University of Minnesota Arnold and Mabel Beckman Center for Transposon Research, Minneapolis, MN 55455, USA; 5 Department of Biochemistry and Molecular Biology, Mayo Clinic, Rochester, MN 55905, USA; 6 Box 45, The Jackson Laboratory, 600 Main Street, Bar Harbor, Maine 04609, USA

## Abstract

**Background:**

The zebrafish is a powerful model vertebrate amenable to high throughput *in vivo *genetic analyses. Examples include reverse genetic screens using morpholino knockdown, expression-based screening using enhancer trapping and forward genetic screening using transposon insertional mutagenesis. We have created a database to facilitate web-based distribution of data from such genetic studies.

**Description:**

The MOrpholino DataBase is a MySQL relational database with an online, PHP interface. Multiple quality control levels allow differential access to data in raw and finished formats. MODBv1 includes sequence information relating to almost 800 morpholinos and their targets and phenotypic data regarding the dose effect of each morpholino (mortality, toxicity and defects). To improve the searchability of this database, we have   incorporated a fixed-vocabulary defect ontology that allows for the organization of morpholino affects based on anatomical structure affected and defect produced. This also allows comparison between species utilizing Phenotypic Attribute Trait Ontology (PATO) designated terminology. MODB is also cross-linked with ZFIN, allowing full searches between the two databases. MODB offers users the ability to retrieve morpholino data by sequence of morpholino or target, name of target, anatomical structure affected and defect produced.

**Conclusion:**

MODB data can be used for functional genomic analysis of morpholino design to maximize efficacy and minimize toxicity. MODB also serves as a template for future sequence-based functional genetic screen databases, and it is currently being used as a model for the creation of a mutagenic insertional transposon database.

## Background

Advances in scientific technology and the availability of genomic sequence for an increasing number of species have led to a paradigm shift from identifying sequence to gene product function. Large functional genetic screens are becoming increasingly prevalent and require a new way of thinking about data to facilitate cross-species comparison and deciphering gene product function in different organisms. Zebrafish are particularly amenable to large screens as they have a tremendous ability to reproduce, and their embryos finish the development of most organ rudiments by 2 days post-fertilization [1]. Their semi-transparent embryos and the ready availability of transgenic lines facilitate functional annotation of the many genes required for numerous organ systems and pathways. While historically their most common use in genetic screenings has been primarily restricted to ENU mutagenesis screens [2-4] and retroviral mutagenesis [5], the availability of morpholinos [6], antisense knockdown oligonucleotides, and insertional mutagenic transposons [7] have opened new avenues for functional genetic research.

Recently, several morpholino screens have been published in collaboration with the Ekker laboratory [8,9]. The largest among them, the Secretome Screen [9,10], identified likely secreted genes from available transcriptome databases using a bioinformatics approach. The Vertebrate Secretome DataBase (VSDB) [4] was established to identify co-translationally translocated (CTT) proteins in several model organism species [10]. Using a combination of signal sequence, transmembrane domain, and initiation site predictors as well as homology to known secreted proteins, a subset of zebrafish CTT genes were identified (0.3× genome coverage based on zebrafish loaded and predicted protein sequences in the CTTome database and Genescan gene prediction estimates of the zebrafish genome [4,5]). 150 gene targets were selected at random for morpholino design, carried out using an Assisted MOrpholino Design tool (AMOD) [11,12]. Each gene was knocked down in zebrafish via morpholino-injection then screened for defects in general morphology and several organ systems including ocular, renal, pigment, vascular, hematopoietic, cranio-facial, and motor [9]. A second morpholino knockdown screen, the Hematopoietic Stem Cell Screen, was conducted using zebrafish homologues of human genes differentially expressed in hematopoietic stem cell differentiation by microarray analysis [8].

Currently, the zebrafish community stores published and sequence-related results on several web-based databases. The principal database for zebrafish-related research is the Zebrafish Information Network (ZFIN) [13,14]. Ensembl [5], likewise, stores sequences targeted by published morpholinos. Ensembl is limited in its ability to store and display experimental data, and while ZFIN contains a wealth of information on published results it lacks specific information such as dosage and searchable phenotype data for published morpholinos and phenotypic data for largely unpublished morpholinos.

Many model organism communities have developed online databases to access results from mutagenic, knock-down/out and over-expression studies. While mouse knockout mutant [15,16] and *C. elegans *RNAi [17,18] experimental result databases with phenotypic content exist, the only morpholino-specific database available is for a pilot *Xenopus *morpholino screen in which 202 genes were targeted and subsequent phenotypes displayed on a webpage [19,20]. The *Xenopus tropicalis *Morpholino Screen database is a flat format website that allows for records to be accessed and viewed by gene targeted, synphenotype group or specific defect. Notably this database does not include search functions and does not offer a means for further input by outside sources. However, the intent of this database as stated by the authors is to display the results from their specific screen [19,20].

Given the intent of our database to display the large amount of data generated from our on-going collaborative screens, we have developed of a scalable, centralized yet easily accessible worldwide, MOrpholino DataBase (MODB) [3] to store and facilitate retrieval of zebrafish morpholino screening results. Additional access has been provided by active links between MODB and ZFIN.

MODB is a scalable, searchable and readily adaptable online MySQL database with a PHP interface. MODB includes storage and retrieval of results generated by our screens and public access of published and unpublished information on morpholinos MODB not only allows dissemination of target-specific information such as effect of knockdown, but also facilitates analysis of morpholino-specific data such as toxicity, dosage, mortality and design. MODB is a new, online, publicly accessible database for data-mining zebrafish morpholino knockdown effects and morpholino non-specific effects.

## Construction and Content

### Construction

**Figure 1:**
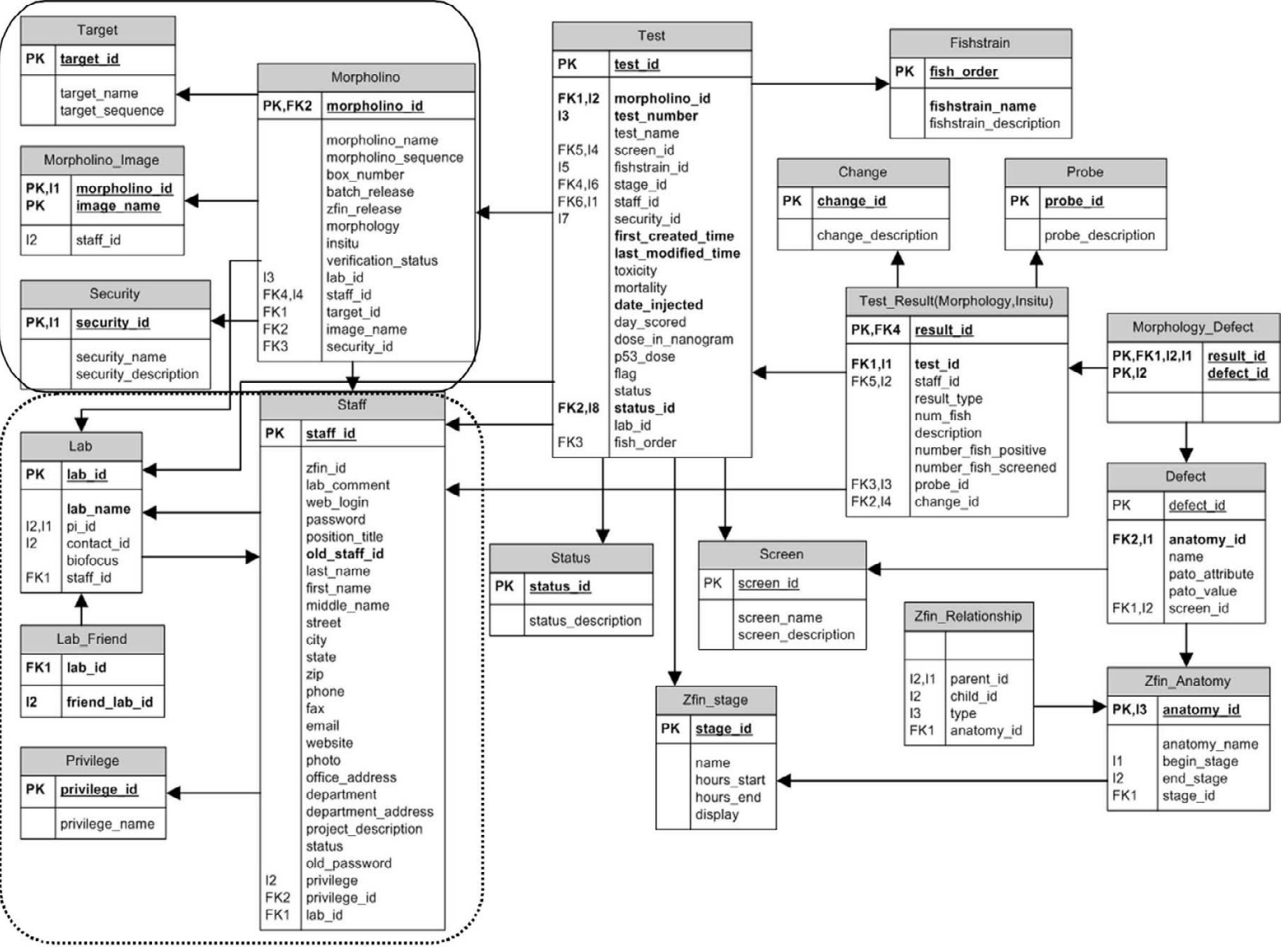
**Entity-Relationship diagram of MODB**. The organization of data contained within MODB is centered on the unique morpholino sequence. Tables pertaining to the morpholino-specific information are located in the upper left corner (solid box). The tables in the lower left corner establish the relations between laboratories, users and security level designation (dashed line box). The tables on the right half of the diagram represent the tests and test components such as anatomical structure, screen and defect observed (unboxed area).

The MODB web project is currently running on an Apache web server [21] with PHP [1] on a Linux system (Intel Xeon, 2.8 GHz, CentOS release 4.4). The project architecture was developed according to the Model-View-Controller (MVC) design paradigm. The Model (database tables) is stored in a MySQL relational database [22]. Major tables include Morpholino, Staff, Lab, Test, and Test_Result and store all information pertinent to each morpholino and users (Figure 1). The View was developed with the help of Smarty [23], a template engine in PHP that helps the designer make modifications in the layout without breaking PHP logic. PHP scripts control the connections between the Model and the View. The GD graphics library (a PHP module) [24] is used for the graphic functions displayed in the summary page.

NCBI BLAST 2.2.13 [25] is invoked by PHP through system calls in the sequence search functions. The MODB BLAST target database is updated automatically when a new morpholino sequence is entered into the database. The ZFIN anatomy ontology [26] was downloaded (release date 08/16/2007) from the ZFIN web site in the OBO format [26] and transferred into the database. An anatomy browser was written in PHP and incorporated in MODB.

### Content

**Figure 2:**
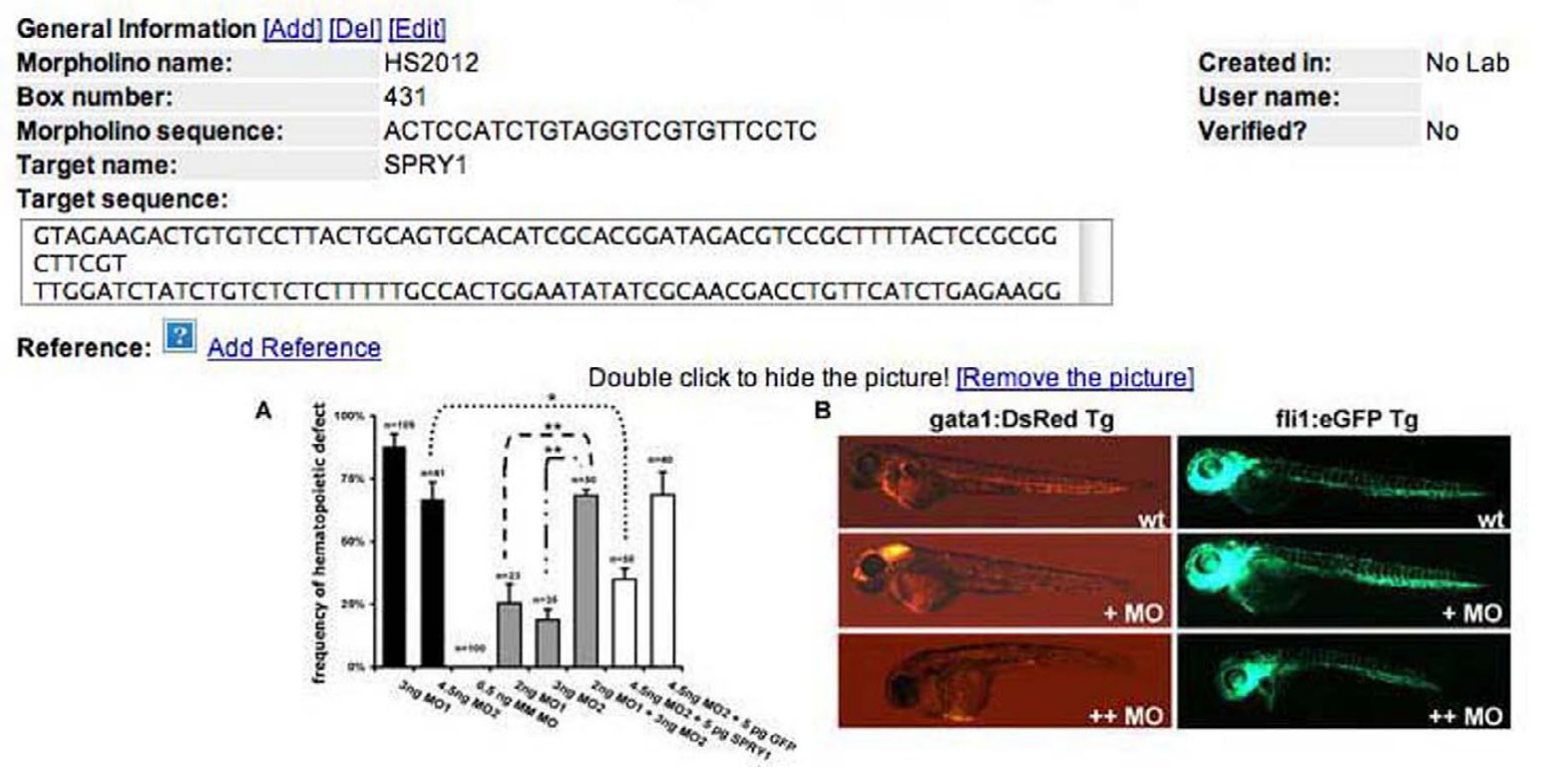
**Morpholino-specific information**. Information such as morpholino name and sequence, in laboratory morpholino designator (box number), creating laboratory, initiating user and any reference images associated with the morpholino are displayed on the initial morpholino page for each morpholino.

The unique identifier in MODB is the sequence of each morpholino. Many morpholino targets in our screens are derived from transcriptome sequence data from The Institute for Genomic Research (TIGR) [27] and GenBank [28] that are subject to change as new sequence data becomes available. For every unique morpholino entered into the database, there is an accompanying set of tables devoted to information pertaining to that morpholino such as name, designator, laboratory created in, contact person, target name, target sequence, gene targeted, reference images and number of tests created (Figure 2). This concise format allows key information regarding the morpholino to be viewed at once.

Early in 2007, MODB contained over 700 unique morpholino sequences generated from several screens, individual projects and control morpholinos. The first generation Secretome Screen [9] is the largest overall contributor of morpholino sequences. From 150 candidate genes sequences, 209 morpholinos were designed. Each morpholino was subjected to a variety of morphological and molecular assays to determine the effect of down-regulating a particular gene product during the first 5-days of zebrafish development [9]. This Secretome Screen identified 26/150 gene targets that produced reproducible effects on specific aspects of early development in early morphology and a variety of organ systems including visual, renal, cardiovascular, cranio-facial, hematopoiesis, and pigment. The more focused Hematopoietic Stem Cells Screen identified 16/64 targets as being necessary for proper blood development [8]. The bulk of the data entered into MODB (124/150 target gene morpholinos in the Secretome Screen and 48/64 in the Hematopoietic Stem Cell Screen) consists of morpholinos that had no observed developmentally specific effect in our screening tests, caused toxic effects such as a high amount of cell death and/or mortality, or produced pleiotropic effects we could not decipher. This information is of use to the scientific community for optimizing morpholino design and learning dosage data and overall effect of previously designed and tested morpholinos.

Since MODB is a living database, new data is regularly being submitted. In the coming year another 50 to 150 morpholinos will be added to the database for the continuing Hematopoietic Stem Cell and secretome screens. To allow the release of data in defined formats and quality, MODB has three data sharing quality control levels. Users are capable of accessing and modifying data related to their designated morpholinos. To facilitate collaborative efforts, a shared designation has been developed to allow members of consortium laboratories to access data related to morpholinos designed by any user within the laboratories that are connected by a relationship within MODB. The third designation ('public') allows morpholino data to be used to generate the summary reports available to the open scientific community via the MODB website. Nearly all morpholinos (779/784) in MODB, with the exception of non-zebrafish morpholinos, are designated as public in MODBv1.

## Utility

### Data Entry

A major function of MODB is data compilation of morpholino-specific information generated by primary research. There are two main ways to add data. Currently, administrators alone enter new listings that provide basic information on a morpholino. Authorized users can submit morpholino sequence and results from specific morphological or molecular experiments for a given observation point and dose. Researchers can apply for an authorized-user identification and password (see author contact information).

**Figure 3:**
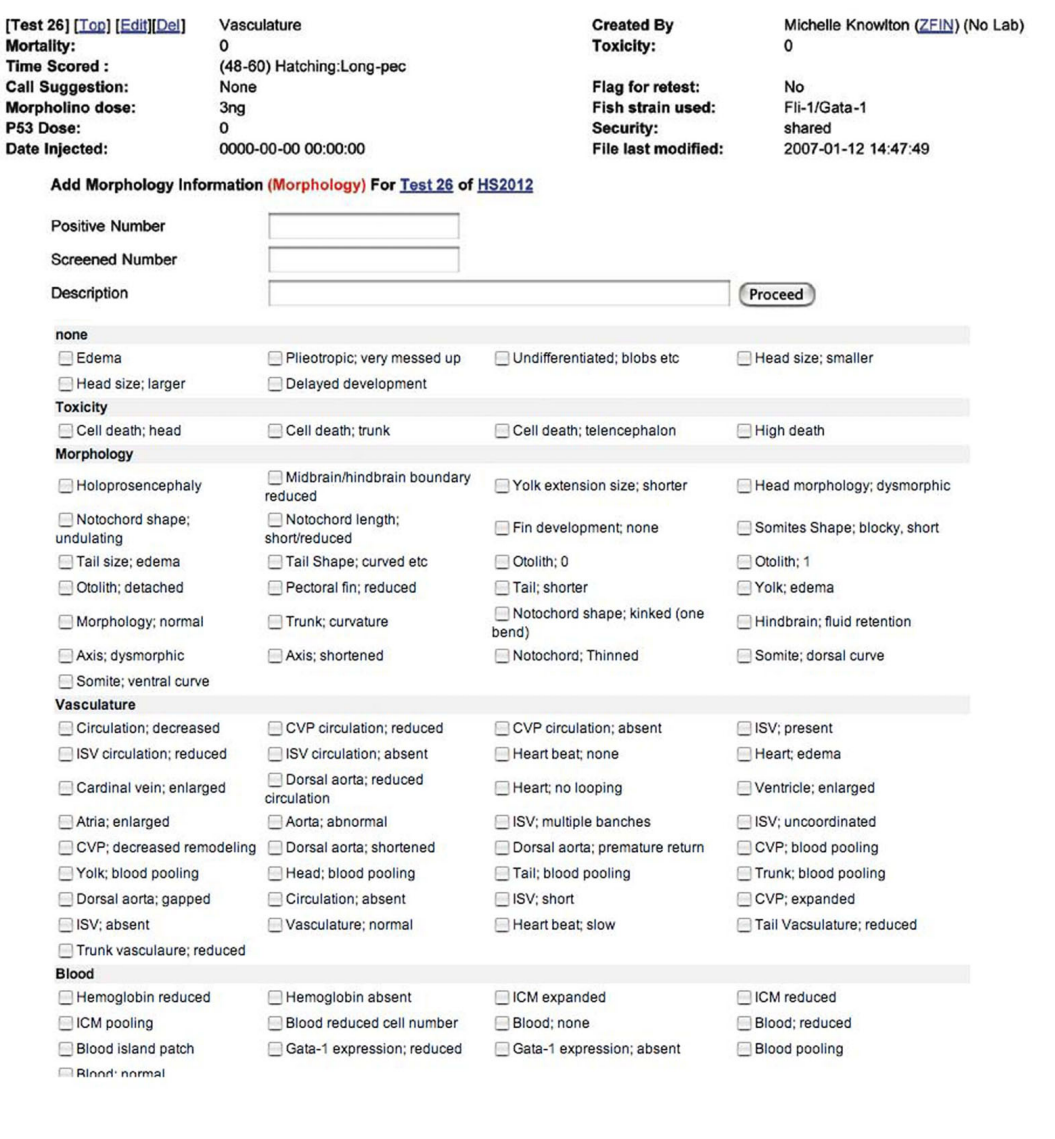
**Common name defect check box**. Entry of observations following morpholino injection into zebrafish embryos is catalogued using a fixed vocabulary checkbox form. Penetrance of observed defect is also recorded for subsequent analysis.

**Figure 4:**
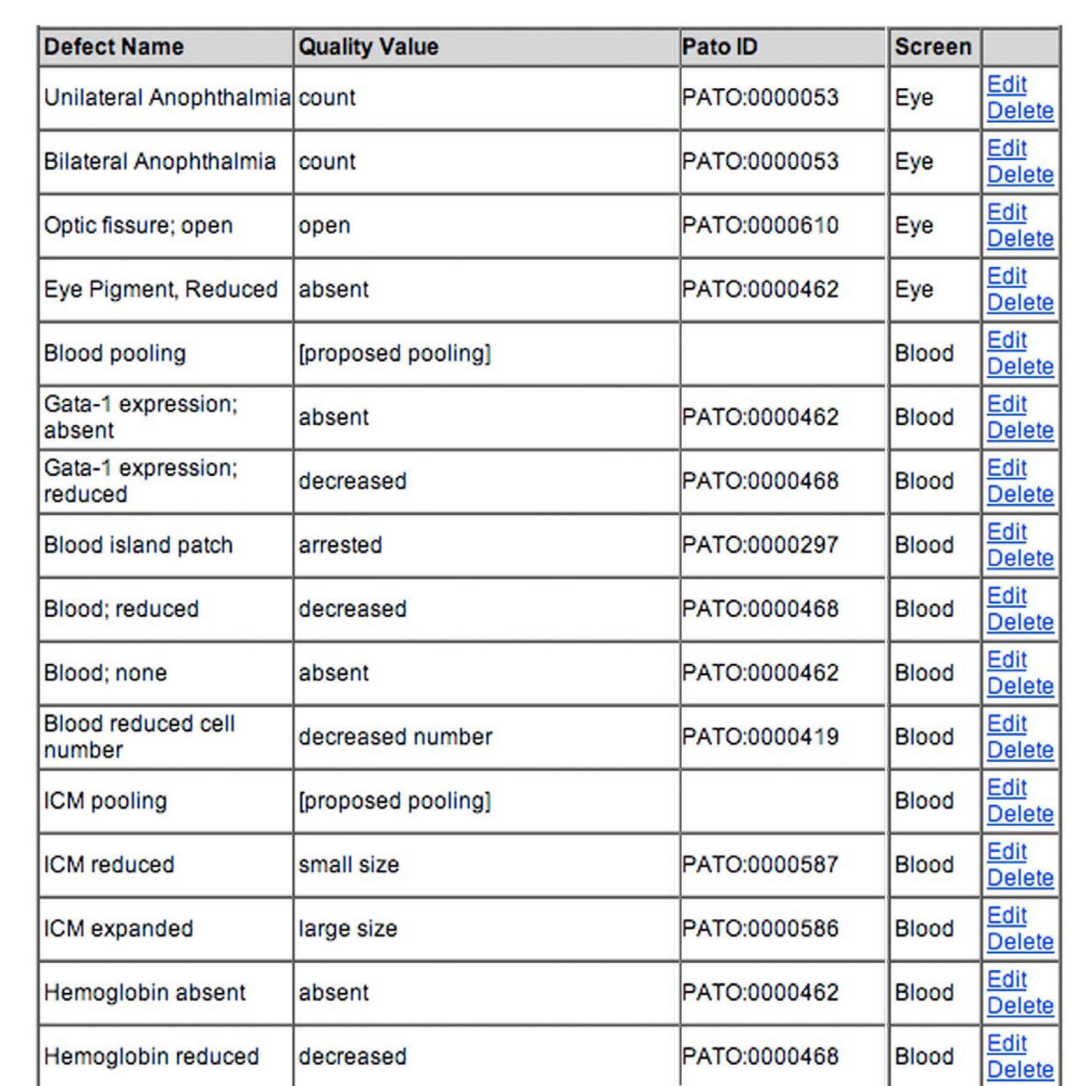
**Correspondence between defect description and PATO designation**. As work is still ongoing on establishing a cross-species phenotypic vocabulary PATO, terminology is constantly evolving. Defect names are being used to maintain some consistency but are rooted in PATO designations via a translation table. When no PATO term exists to adequately describe the defect observed, a proposed term is submitted to NCBO via ZFIN.

These results are called 'tests'. In early 2007, MODB contained over 9000 individual test entries. A free text notes field permits detailed description defects observed in morpholino-injected embryos once a fixed vocabulary defect term has been selected. The defects ontology check box was established with common scientific terms used in our screening protocols (Figure 3). Specific in-laboratory scientific descriptions correspond to established defects ontology. The reduction of free text and the promotion of fixed vocabularies increases the searchability of MODB for search by keyword, anatomical structure or fixed defects term and allows for future integration with other fixed-vocabulary phenotype databases such as WormBase and the Mouse Phenome database [15,29]. To utilize the data entered into MODB in a meaningful manner, we developed a variety of search functions. In addition to the parameters already mentioned (laboratory designator, morpholino name, morpholino target name, gene targeted, laboratory created in, or keyword), MODB can be queried by a controlled defects vocabulary. This defects ontology was developed in conjunction with ZFIN to model the current status of Phenotypic Attribute Trait Ontology (PATO) developed by the National Center for Biomedical Ontology (NCBO) [30]. To facilitate the use of this common language, MODB users assess phenotypes using defect descriptions linked to current PATO terminology (Figure 4). Initially, PATO was designed to describe a defect observed in an anatomical structure (the entity, e.g. tail) using a trait (e.g. relative length) and attribute of that trait (e.g. short length). This system has subsequently been replaced with an entity and quality designation (e.g. tail, short length). MODB's defects ontology contains the latter version of PATO. When no PATO term exists to adequately describe the defect observed, a proposed term is submitted to NCBO via ZFIN to assist in the development of a cross-species phenotypic ontology. The importance of a common phenotypic vocabulary resides in the ability to perform cross-species analysis of gene function and conserved pathways.

### Data retrieval

**Figure 5:**
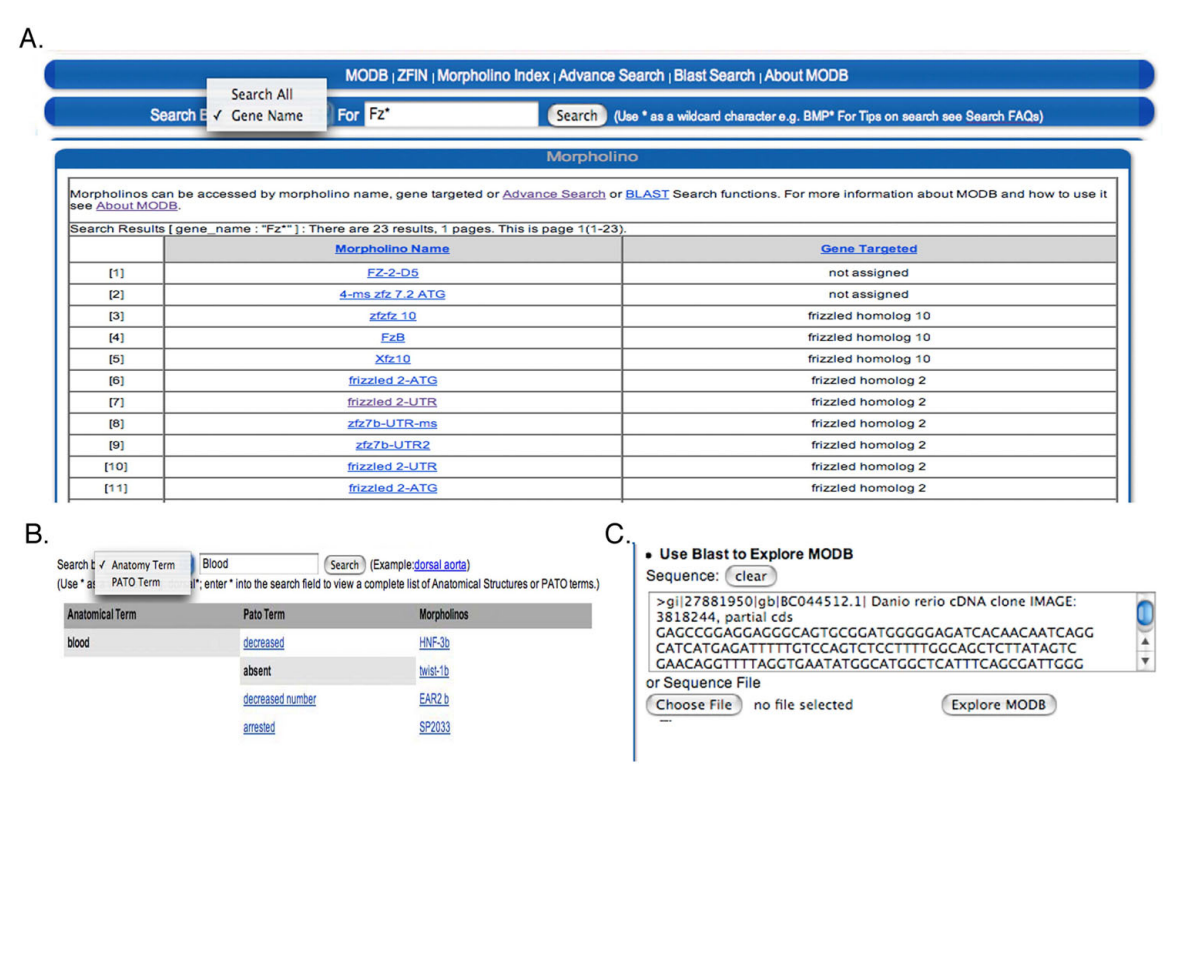
**MODB search functions**. **A**. Data retrieval from MODB is simplified by morpholino name and gene targeted. Results can be sorted morpholino name and gene targeted. **B**. An additional search in the public interface allows for searches based on PATO term and anatomical structure affected. **C**. An additional sequence-based search using morpholino or target sequence can be performed.

The usefulness of the data stored within any database is subject to the retrieval methods available. MODB has been designed with numerous search and sort functions that allow for retrieval by morpholino sequence, morpholino name and gene targeted (Figure 5A). An advanced search feature in the public interface allows users to search by anatomy and phenotype to identify morpholinos with a given defect (Figure 5B). A sequence-based search is also available, using either the target morpholino sequence or the sequence of the morpholino (Figure 5C). By offering numerous search and sorting options, results of nearly 800 morpholinos and more that 9000 tests can be accessed and analyzed for further scientific insight into the knockdown effect of a specific morpholino target, genes effecting the same developmental pathways, and non-specific morpholino effects.

### MODB v1 Public Interface

**Figure 6:**
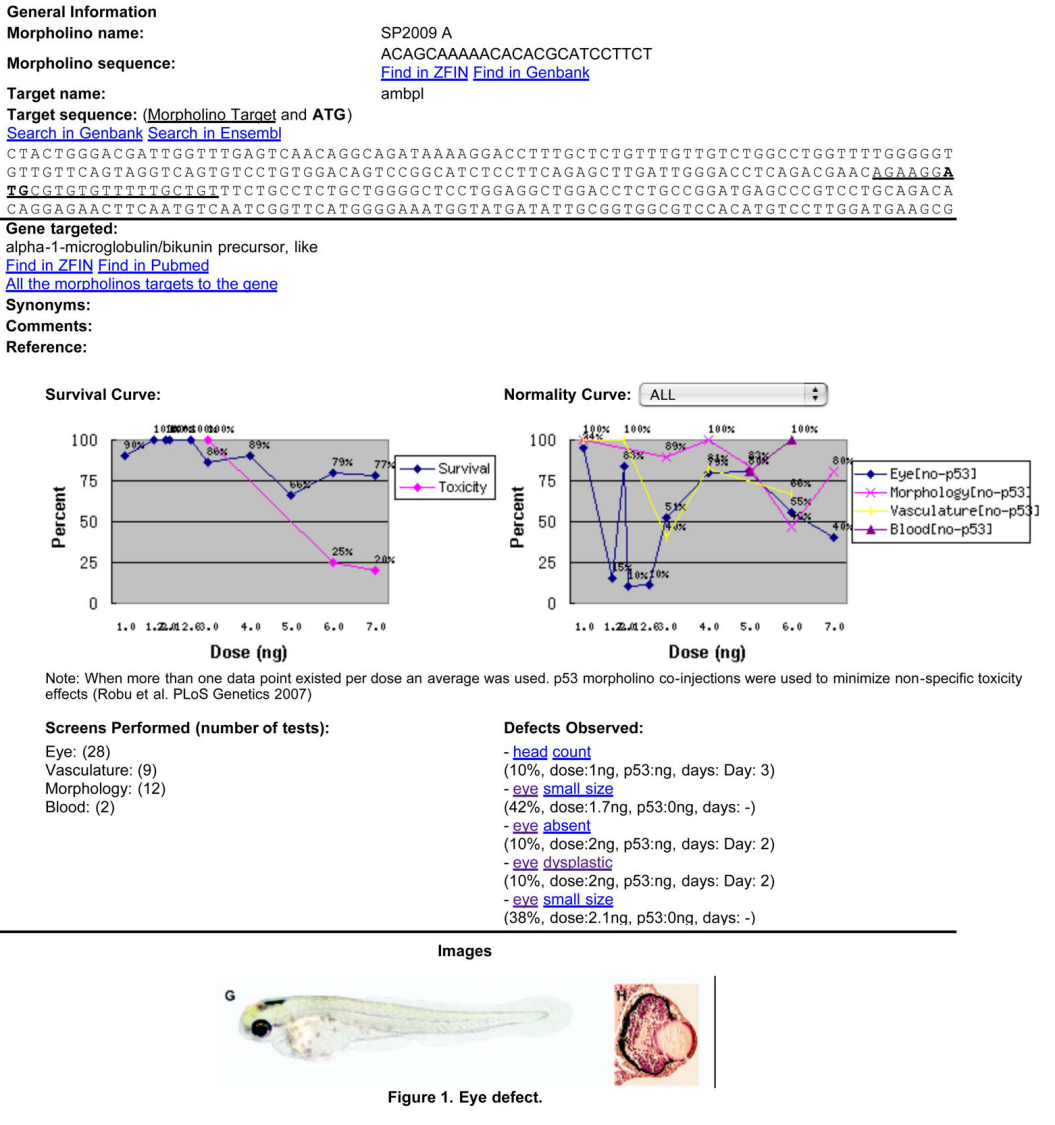
**Public summary page**. A publicly accessible summary page is available for released morpholinos in MODB. This page contains morpholino and target information such as name, sequence etc. and a review of knockdown results such as dosing for mortality and normalcy. A list of tests performed, defects observed and images is also displayed.

To facilitate display and digestion of the large amount of data stored within MODB, a summary sheet for each morpholino was created (see construction and content) and is publicly available. Each morpholino summary contains sequence and target information as well as survival and normalcy (percent normal embryos observed overall or for specific screen types) curves based on dose (Figure 6). Several search functions can be used to access this information including by gene targeted, BLAST and an advanced search function that facilitates searching for morpholinos by PATO designation and anatomical structure affected (Figure 5B). MODB thus presents users with the valuable knowledge, such as mortality and dose curves, effectiveness and morphant phenotype. Currently, 101 morpholinos contain hyperlinks to specific morpholino entries in ZFIN and hyperlinks are provided for gene targets in MODBv1.

## Discussion

With advent of whole-genome sequencing technology, a greater emphasis on gene function is emerging. Functional genomic screening tools available in zebrafish such as ENU mutagenesis, GripNAs, RNAi, insertional mutagenesis and morpholinos allow for large functional gene screens that produce an enormous amount of biological information (reviewed in [2,6,7,31-33]). Efficient data storage is critical to allow for retrieval of results in a meaningful fashion and to facilitate data mining. MODB is a non-proprietary, relational database with online accessibility that acts as a storehouse for critical information relating to sequence-specific morpholino knockdown screens. MODB is, to our knowledge, unique among the available databases in that it offers searchable morpholino-specific data and fixed-vocabulary defects ontological cataloging of morphants that allows for cross-species analysis using a common, PATO-derived ontology. Cross-linking to ZFIN provides additional value of MODB for the research community.

Continued screening efforts in zebrafish using morpholinos and other sequence-specific knockdown or knockout technologies will expand our knowledge of gene function. MODB provides a central repository for morpholino information. This schema is potentially applicable to any sequence-based screening approach in zebrafish or related systems. For example, MODB is currently being adapted to the creation of a transposon database that includes insertional mutagenesis and expression analysis data.

## Conclusion

By offering public access to our schema and the data contained within MODB, we hope to aid in the advancement of functional genetic studies and gene annotation as well as improvements in morpholino analysis and design. The dissemination of valuable knowledge gained via large screens such as the Secretome [9] and Hematopoietic Stem Cell [8] screens goes beyond published gene target characterization and provides a foundation for future research.

## Availability and requirements

MODB is accessible via the web . To be added as an authorized user, contact the corresponding author M.K. or S.C.E. Morpholinos are readily available for purchase through Gene Tools [34] or Open Biosystems [35]. The authors have no affiliation, financial or otherwise, with either corporate entity.

## Authors' contributions

MK designed, tested and managed the database. TL and YR were responsible for the programming, debugging and informatics support. BB aided in the conception, design and testing of the database. EL and SE aided in the conception and design of the database and coordinated the project as a whole. All authors read and approved the final manuscript.
